# The bounce of the body in hopping, running and trotting: different machines with the same motor

**DOI:** 10.1098/rspb.2009.1317

**Published:** 2009-09-16

**Authors:** G. A. Cavagna, M. A. Legramandi

**Affiliations:** Dipartimento di Fisiologia Umana, Università degli Studi di Milano, 20133 Milan, Italy

**Keywords:** locomotion, running, hopping, trotting, landing–takeoff asymmetry, muscle force–velocity relation

## Abstract

The bouncing mechanism of human running is characterized by a shorter duration of the brake after ‘landing’ compared with a longer duration of the push before ‘takeoff’. This landing–takeoff asymmetry has been thought to be a consequence of the force–velocity relation of the muscle, resulting in a greater force exerted during stretching after landing and a lower force developed during shortening before takeoff. However, the asymmetric lever system of the human foot during stance may also be the cause. Here, we measure the landing–takeoff asymmetry in bouncing steps of running, hopping and trotting animals using diverse lever systems. We find that the duration of the push exceeds that of the brake in all the animals, indicating that the different lever systems comply with the basic property of muscle to resist stretching with a force greater than that developed during shortening. In addition, results show both the landing–takeoff asymmetry and the mass-specific vertical stiffness to be greater in small animals than in large animals. We suggest that the landing–takeoff asymmetry is an index of a lack of elasticity, which increases with increasing the role of muscle relative to that of tendon within muscle–tendon units.

## 1. Introduction

A landing–takeoff asymmetry has been described in the apparently elastic bounce of the body during human running. In each bounce, some of the mechanical energy of the centre of mass of the body is absorbed by muscle–tendon units during the brake and successively restored during the push. In a symmetric elastic system, the duration of the brake equals that of the push. This was found not to be true in human running. The rebound is asymmetric: at low and intermediate running speeds the duration of the push exceeds that of the brake (Cavagna 2006; Cavagna *et al*. 2008).

During running on the level at a constant speed, the momentum lost in the sagittal plane during the brake equals the momentum gained during the push. Since momentum is the product of force and duration of force application, the greater duration of the push implies that the force developed during the push, when muscle–tendon units shorten, is smaller than that exerted during the brake, when muscle–tendon units are stretched. In other words, the force performing negative work during the brake of the body after landing is greater than the force performing positive work during the push before takeoff. A force during negative work greater than during positive work is consistent with the force–velocity relation of muscle contractile component (i.e. with the basic property of muscle to resist stretching with a force greater than that developed during shortening). Therefore, the landing–takeoff asymmetry in human running has been considered to be a consequence of the asymmetry of the force–velocity relation of muscle (Cavagna 2006).

The force–velocity relation of muscle however is not the only candidate to be considered as a cause of the landing–takeoff asymmetry. As Professor McNeill Alexander pointed out in conversation (personal communication, 5 July 2006), the different length of the moment arms between heel and ankle compared with between ankle and toe during stance should be taken into account as a possible explanation of the landing–takeoff asymmetry in human running. In fact, locomotion results from the interaction of a motor (the muscular system) and a force-transmission machine (the skeletal lever system). Muscle transforms chemical energy into mechanical work, which is then used by the lever system to promote forward movement of the body. The absolute amount of negative work (during the brake) equals that of positive work (during the push) when running on the level at a constant speed. Since work is force times displacement, the greater force developed during the brake implies a displacement of the centre of mass of the body in the sagittal plane smaller during negative work than during positive work. This is what one may expect from the asymmetric lever system of the human foot, since the moment arm between heel and ankle, operating after landing (brake), is shorter than the moment arm between ankle and toe, operating before take-off (push; Carrier *et al*. 1994). The following alternative hypothesis could therefore be made to explain the landing–takeoff asymmetry of running. The force during the brake is greater than during the push because the displacement of the centre of mass during negative work is smaller than that during positive work due to the asymmetric lever system. In other words, the greater force exerted during the brake may be required to cope with the smaller displacement at disposal during negative work. From this point of view, the difference in force may not be the consequence of the force–velocity relation of muscle, but would be the consequence of the asymmetric lever system, which would then be the initial cause of the landing–takeoff asymmetry. The question therefore arises: what is the cause of the landing–takeoff asymmetry in human running? The motor, the machine or both?

It has been shown that the bouncing mechanism of running initially described for humans (Cavagna *et al*. 1964) also applies to hopping mammals, running birds and trotting quadrupeds (Cavagna *et al*. 1977). These animals use a machine (lever system) to promote locomotion which differs from that of humans. In particular, hopping kangaroos and springhares, running birds and trotting quadrupeds land with the digits first, contrary to humans who commonly run with a heel strike. In this study, we re-analyse the bounce of the body in these animals to determine the effect of their different lever systems on the landing–takeoff asymmetry.

We hypothesize that a persistence of the landing–takeoff asymmetry found in humans would indicate that the different machines comply with the basic property of muscle to resist stretching with a force greater than during shortening. In addition we relate the landing–takeoff asymmetry to the mass-specific vertical stiffness measured in animals of different sizes exhibiting different step frequencies and different natural frequencies of their bouncing system.

## 2. Material and Methods

### (a) Subjects and experimental procedure

We re-analysed 136 records, previously obtained by Cavagna *et al*. (1977), of the vertical and forward velocity changes of the centre of mass of the body during hopping, running and trotting on a force platform sensitive to the vertical and forward component of the force exerted by the feet on its surface. The vertical and forward velocity changes were obtained during the experiments of Cavagna *et al*. (1977) by analogical integration of the platform signals made simultaneously with the animal run. Data in table 1 derive from measurements made in 31 records of two kangaroos (*Megaleia rufa*, about 20 kg each) and 26 of a springhare (*Pedetes cafer*, 2.5 kg) during hopping; 12 records of two wild turkeys (*Meleagris gallopavo*, about 7 kg) and 13 of a rhea (*Rhea Americana*, 22.3 kg) during running; and 28 records of two dogs (*Canis familiaris*, weighing 5 and 17.6 kg), 14 of a monkey (*Macaca speciosa*, weighing 3.1 kg) and 12 of two rams (*Ovis musimon*, weighing approx. 60 and 85 kg) during trotting. The characteristics of the platform and the principle of the method followed to process the platform's signals are described in detail in previous studies (Cavagna 1975; Cavagna *et al*. 2008). Analytical procedures used in this study are described briefly below.

**Table 1.** Values are mean ± s.d. of measurements made in all the runs of figure 2. The number of items in the mean *n* is given below the name of each animal. From top to bottom: the mass of the animal; the positive work duration, *t*_push_; the negative work duration, *t*_brake_; the landing–takeoff asymmetry, *t*_push_/*t*_brake_ (mean of the ratios made at each speed); the mass-specific vertical stiffness, *k/M*_b_; the natural frequency of the bouncing system, *f*_s_ = (1/2π)√*k*/*M*_b_; the step frequency, *f*_step_; the maximal upward acceleration, *A*_v,mx,up_; and the efficiency of doing external work, *W*_ext_/*W*_metab_. Note that (i) *t*_push_ is greater than *t*_brake_ (*p* < 0.05 in all animals except rams where *p* = 0.13); (ii) *t*_push_/*t*_brake_ is greater in small animals than in large animals (*p* < 0.05 in all animals except rams versus monkey where *p* = 0.09); and (iii) *k/M*_b_ is greater in small animals than in large animals (*p* < 0.05).

	hop		run		trot			
	kangaroos (*n* = 31)	springhare (*n* = 26)	rhea (*n* = 13)	turkeys (*n* = 12)	large dog (*n* = 12)	small dog (*n* = 16)	rams (*n* = 12)	monkey (*n* = 14)
mass (kg)	20.4 ± 0.5	2.5 ± 0.1	22.4 ± 0.4	7.2 ± 0.3	17.6 ± 0.2	5.0 ± 0.2	76.2 ± 11.4	3.1 ± 0.2
*t*_push_ (s)	0.095 ± 0.010	0.069 ± 0.012	0.118 ± 0.019	0.102 ± 0.017	0.128 ± 0.014	0.093 ± 0.012	0.158 ± 0.033	0.103 ± 0.017
*t*_brake_ (s)	0.088 ± 0.013	0.059 ± 0.012	0.094 ± 0.014	0.071 ± 0.014	0.105 ± 0.014	0.071 ± 0.011	0.149 ± 0.025	0.091 ± 0.023
*t*_push_/*t*_brake_	1.091 ± 0.151	1.190 ± 0.196	1.272 ± 0.189	1.443 ± 0.140	1.217 ± 0.099	1.334 ± 0.145	1.062 ± 0.119	1.166 ± 0.182
*k*/*M*_b_ (s^−2^)	644.9 ± 170.5	1411.3 ± 492.0	596.7 ± 270.0	886.7 ± 252.7	762.1 ± 182.0	1547.4 ± 378.2	397.2 ± 183.3	1193.0 ± 444.4
*f*_s_ (Hz)	4.01 ± 0.52	5.90 ± 1.02	3.82 ± 0.77	4.70 ± 0.65	4.36 ± 0.55	6.21 ± 0.80	3.10 ± 0.68	5.41 ± 1.00
*f*_step_ (Hz)	2.19 ± 0.19	3.04 ± 0.20	3.63 ± 0.43	4.44 ± 0.43	4.31 ± 0.41	6.18 ± 0.69	3.31 ± 0.64	5.21 ± 0.87
*A*_v,mx,up_ (ms^−2^)	39.45 ± 4.72	42.30 ± 10.85	12.53 ± 2.64	13.40 ± 2.58	10.44 ± 1.61	8.99 ± 1.10	6.19 ± 2.41	9.66 ± 2.59
*W*_ext_/*W*_metab_	0.501	0.207	0.147	0.124	0.262	–	–	0.153

### (b) From velocity changes to mechanical energy of the centre of mass

**Figure 1.**
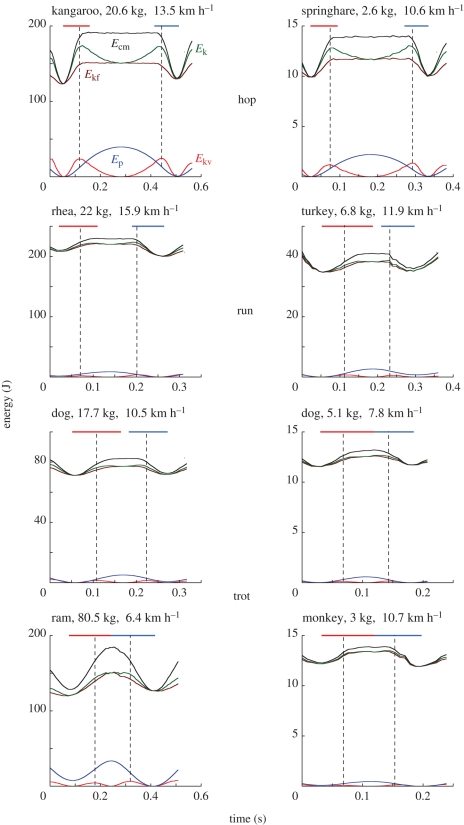
Mechanical energy of the centre of mass of the body during steps of hopping, running and trotting animals at the indicated speeds. Left column, larger animals; right column, smaller animals. In each panel the curves show the gravitational potential energy (*E*_p_, blue), the kinetic energy of vertical motion (*E*_kv_, red), the kinetic energy of forward motion (*E*_kf_, brown), the kinetic energy of motion in the sagittal plane (*E*_k_ = *E*_kv_+*E*_kf_, green) and the total translational energy of the centre of mass of the body in the sagittal plane (*E*_cm_ = *E*_p_*+E*_k_, black). The interrupted vertical lines through the peaks of *E*_kv_ indicate the instants of static equilibrium position when the bouncing system is loaded with a vertical force equal to body weight. The red horizontal bars indicate the time during which positive external work is done, *t*_push_ (increment of *E*_cm_), whereas the blue horizontal bars indicate the time during which negative external work is done, *t*_brake_ (decrement of *E*_cm_). The gap between red and blue bars indicates the duration of the aerial time (when present). The landing–takeoff asymmetry, indicated by *t*_push_ > *t*_brake_, measures the discrepancy from an elastic rebound where *t*_push_ = *t*_brake_.

**Figure 2.**
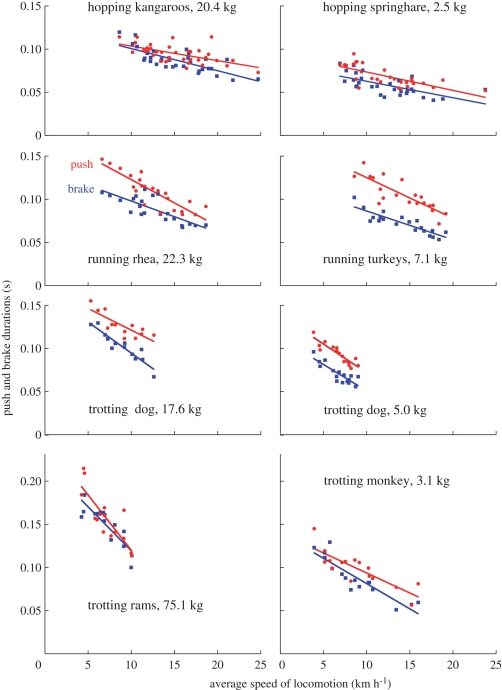
The positive work duration *t*_push_ (red continuous line and circles) and the negative work duration *t*_brake_ (blue continuous line and squares) are plotted as a function of speed for all the runs of the animals whose steps are illustrated in figure 1. Lines (Kaleidagraph 4.03 linear fits) are just a guide for the eye and do not describe the underlying physical mechanism. Note that *t*_push_ is greater than *t*_brake_, suggesting that the different machines promoting locomotion in these animals are similarly affected by the basic property of muscle to develop a lower force during shortening than during stretching.

The velocity change records were interpolated every 8–9 ms (Kaleidagraph v. 4.03) to reduce a high-frequency noise present in the magnetic substrate we recovered. A compromise was searched between the reduction of the noise and the reduction of the points available for the analysis. The interpolated curve was analysed by means of custom LabView software (National Instruments, Austin, TX, USA, v. 7.1) to measure the instantaneous vertical velocity *V*_v_(*t*) and forward velocity *V*_f_ (*t*) of the centre of mass, the kinetic energy of vertical motion *E*_kv_(*t*) = 0.5*M*_b_*V*_v_(*t*)^2^ (where *M*_b_ is the mass of the body), the kinetic energy of forward motion *E*_kf_ (*t*) = 0.5*M*_b_*V*_f_ (*t*)^2^, the gravitational potential energy *E*_p_(*t*) = *M*_b_ *g S*_v_(*t*) (where *S*_v_(*t*) is the vertical displacement of the centre of mass obtained by integration of the vertical velocity, and *g* the acceleration of gravity), and the total mechanical energy *E*_cm_(*t*) = *E*_kv_(*t*) + *E*_kf_ (*t*) + *E*_p_(*t*) (figure 1). The work done at each step against gravity, *W*_v_, to sustain the forward velocity changes, *W*_kf_, and the total mechanical energy changes of the centre of mass, *W*_ext_, were measured from the *E*_p_(*t*), *E*_kf_ (*t*) and *E*_cm_(*t*) records, respectively. Positive values of the energy changes gave positive work, negative values gave negative work. In a perfect steady run on the level, the ratio between the absolute values of positive and negative work done in an integer number of steps should be equal to one. The regularity of the selected steps was therefore assessed from the ratio between positive and negative work. Initially, steps where 0.5 < *W* ^+^/*W* ^−^ < 1.5 were used for analysis. Experimental values were as follows (*n* = 167): *W* ^+^_v_/*W*^−^_v_ = 1.021 ± 0.116 and *W* ^+^_kf_ /*W* ^−^_kf_ = 1.019 ± 0.166 and *W* ^+^_ext_ /*W* ^−^_ext_ = 1.017 ± 0.113. Subsequently, steps where 0.75 < *W* ^+^/*W* ^−^ < 1.25 were used for analysis. Experimental values were as follows (*n* = 136): *W* ^+^_v_/*W* ^−^_v_ = 1.015 ± 0.095, *W* ^+^_kf_ /*W* ^−^_kf_ = 1.010 ± 0.120, *W* ^+^_ext_/*W* ^−^_ext_ = 1.014 ± 0.084. Data in figure 2 and table 1, obtained from measurements made on the 136-record sample, do not differ appreciably from those made on the 167-record sample.

### (c) Measurement of the landing–takeoff asymmetry

As described in §1, the landing–takeoff asymmetry is revealed by the duration of the push, *t*_push_, being greater than that of the brake, *t*_brake_. The ratio *t*_push_/*t*_brake_ was therefore taken as a measure of the landing–takeoff asymmetry (figure 2; table 1). Push and brake durations were measured from the increment and the decrement, respectively, of the total mechanical energy of the centre of mass, *E*_cm_ (black curve in figure 1).

The difficulty in measuring the increment (*t*_push_) and the decrement (*t*_brake_) of *E*_cm_ is mainly due to the blunt attainment of the *E*_cm_ plateau. In order to make this transition more sharp, the derivative d*E*_cm_(*t*)/d*t* was made. This procedure did succeed in making the transition to the *E*_cm_ plateau more clear, but resulted in a noise of the d*E*_cm_(*t*)/d*t* record during the *E*_cm_ plateau itself. Two reference levels on the d*E*_cm_(*t*)/d*t* record were therefore set just above and just below the noise of the d*E*_cm_(*t*)/d*t* record during the *E*_cm_ plateau: the time interval during which the d*E*_cm_(*t*)/d*t* record was above the upper line, without crossing the noise, was automatically taken by the program as *t*_push_, whereas the time interval during which d*E*_cm_(*t*)/d*t* record was below the horizontal line, without crossing the noise, was taken as *t*_brake_ (Cavagna 2006). However, as pointed out by Cavagna (2006), this procedure is inconvenient in underestimating both *t*_push_ and *t*_brake_. In fact a fraction of the time interval during which the d*E*_cm_(*t*)/d*t* record was indeed above and below the ideal line, where d*E*_cm_(*t*)/d*t* ∼ 0, was missed to avoid crossing the noise of the d*E*_cm_(*t*)/d*t* record during the *E*_cm_ plateau. The error is obviously larger the greater the noise, and the noise is larger the smaller the animal. An improvement of the method previously used was therefore applied in this study, by measuring push and brake durations as the time intervals during which the d*E*_cm_(*t*)/d*t* record was respectively above and below the *mean* of the data points comprised between the two reference levels (not above and below the two reference levels as previously made). This procedure requested manual instead of automatic measure of *t*_push_ and *t*_brake_ in 11 of the 136 records analysed. In most trotting steps it was not possible to detect a fraction of the step, where d*E*_cm_(*t*)/d*t* ∼ 0 and the two reference levels were superposed. In this case, both *t*_push_ and *t*_brake_ were automatically measured by the software as the number of data points respectively above and below the superposed reference levels.

### (d) Vertical stiffness

The mass-specific vertical stiffness, *k/M*_b_, is given by the slope of the relationship between vertical acceleration and vertical displacement of the centre of mass in the range corresponding to the amplitude of the oscillation of the spring–mass system, i.e. from its equilibrium position (vertical acceleration = 1*g*) to its maximal deformation (Cavagna *et al*. 1988). In this study we measured the maximal upward acceleration *A*_v,mx,up_ of the centre of mass of the body, attained at the lowest point of its trajectory, and the vertical displacement of the centre of mass *S*_ce_ from the lowest point to the equilibrium position. The vertical acceleration was deduced from the time derivative of the vertical velocity, whereas the vertical displacement was obtained by integration of the vertical velocity. The mass-specific vertical stiffness was measured as *k/M*_b_ = *A*_v,mx,up_ /*S*_ce_. Note that the vertical stiffness as defined here gives an unambiguous measure of the stiffness of the elastic structures only on the assumption that the muscle is kept isometric during the stretch–shorten cycle of muscle–tendon units and the whole of the length change is taken by elastic elements. Note also that the mass-specific vertical stiffness results in a natural frequency of the bouncing system *f*_s_ = (*k*/*M*)^1/2^/(2*π*), for which the connection with the step frequency in trotting, running and hopping (table 1) has been shown in a previous study (Cavagna *et al*. 1988). Furthermore, the peak in stored ‘elastic’ energy is attained at the lowest point of the oscillation of the centre of mass when the system is loaded by the vertical force only, since gravitational potential energy and kinetic energy of forward motion are in opposition of phase during the bouncing step. This energy, however, may also be used to accelerate the body forwards during the lift due to the specific instantaneous orientation of the line connecting the centre of mass with the ground.

### (e) Statistics

The average values reported in this study represent the mean ± s.d. of the data measured over the whole speed range of locomotion in each animal. A paired-sample *t*-test was used to determine when the means of *t*_push_ and *t*_brake_, with the same number of items measured in the same animal and in the same steps, are significantly different. When comparing the means of different variables (*t*_push_/*t*_brake_ and *k/M*_b_) between two subject groups having different numbers of items, a two-sample *t*-test assuming unequal variances was used. The values of *p* in table 1 legend refer to the two-tail comparison (Excel for Mac v. 11.3.5).

## 3. Results

Figure 2 shows the positive and negative work durations measured as indicated in figure 1 (red and blue bars) in hopping, running and trotting steps at different speeds. It can be seen that the duration of positive work *t*_push_ is greater than the duration of negative work *t*_brake_ in all the animals in spite of their different anatomy, body weight and systems of locomotion.

The landing–takeoff asymmetry of the bounce increases with increasing ratio *t*_push_/*t*_brake_, which is given in table 1 together with the mass-specific vertical stiffness of the bouncing system *k/M*_b_. Both *t*_push_/*t*_brake_ and *k/M*_b_ are greater in the animals of smaller size and body weight, suggesting that the landing–takeoff asymmetry of the bounce increases with the stiffness of the bouncing system.

The physiological meaning of the landing–takeoff asymmetry in the animal bounce is evidenced, as described below, by a comparison with a purely elastic rebound of the mechanical energy attained by the centre of mass *E*_cm_, during its downward and upward displacement, at the two points where *E*_kv_ is at a maximum (vertical dotted lines in figure 1). These two points correspond to a condition of static equilibrium of the spring–mass system loaded with a vertical force equal to body weight (Blickhan 1989) and can be conveniently used as reference points for a comparison with an elastic system.

In the elastic rebound of a spring–mass system, the mechanical energy of the centre of mass at the equilibrium position during the descent equals the mechanical energy of the centre of mass at the equilibrium position during the lift. This is because the kinetic and gravitational potential energy of the centre of mass is stored during the descent as elastic potential energy and converted without loss back into kinetic and gravitational potential energy attaining the same value during the lift. Figure 1 shows that this condition is approached in the bounce of a kangaroo.

The rebound of the body in the animals showing a large landing–takeoff asymmetry, i.e. a large ratio *t*_push_/*t*_brake_, differs drastically from an elastic rebound. Consider, for example, the running turkey in figure 1, which shows the greatest landing–takeoff asymmetry. The intersection of the interrupted lines with the *E*_cm_ curve in figure 1 shows that the mechanical energy during the lift is less than the mechanical energy during the fall, indicating that some losses occur in the stretch–shorten cycle of muscle–tendon units. During the stretch, these losses are expected to occur due to cross-bridge detachment if muscle, instead of tendon, is forcibly lengthened (i.e. if tendons are stiffer than muscle). Due to these losses, some energy must be added to complete the lift of the centre of mass and to accelerate it forwards to the velocity attained before the brake. This additional energy must derive from the active muscular contraction, which, according to the force–velocity relation of the contractile component, is characterized by a lower force developed during shortening. This lower force necessarily requires more time to restore the momentum lost during stretching when the force is higher, thus explaining why *t*_push_ > *t*_brake_. According to this analysis, therefore, the landing–takeoff asymmetry is a measure of the lack of elasticity in the rebound of the body and is expected to increase with increasing contribution of muscle relative to tendon in the stretch–shorten cycle of muscle–tendon units. In fact contracting muscle exhibits a large hysteresis in its stretch–shorten cycle, whereas a very low hysteresis is found in the stretch–shorten cycle of tendons (Alexander 2002).

The ‘elastic’ mechanism suggested above may not be the only cause of the landing–takeoff asymmetry in the animal bounce. Given the large number of limb and trunk muscles that produce, absorb and re-distribute energy within the limbs and body during the bounce, it is possible that antagonistic work done by muscles against others may contribute to the observed landing–takeoff asymmetry.

## 4. Discussion

### (a) Different machines with the same motor

Locomotion is carried on in the animals of this study and in humans with a large diversity in the anatomy and geometry of their machines (i.e. of the lever systems, which promote forward movement of the body). The different machines serve different tasks and are used differently during the step. For example, whereas in human running landing takes place on the heel and takeoff from the front of the foot, in running in birds landing takes place on the front of the foot far from the ankle, which is shifted upwards relative to the ground. Thanks to this geometry the digits of birds, instead of the knee, occupy a front position when the legs are flexed against the body during the flight and are ready to grasp support at landing. In hopping and trotting also, contact with the ground takes place with the front of the foot. In hopping, a long duration of the aerial phase is required to allow repositioning of the same two feet over which each bounce takes place, whereas in trotting a minimal, often absent, aerial phase is inserted between bounces on two (front–back) feet of opposite sides of the body. The characteristics of the environment may also modify the anatomy of the locomotor's machine. Differences in hind limb anatomy and in hopping mechanics have been found in two species of wallabies inhabiting different environments (McGowan *et al*. 2008). Other examples could be given showing how different machines evolved differently in order to fulfil different requirements in different surroundings and are used differently during locomotion because of their different geometries and structure.

In contrast with the large diversities mentioned above, the motor operating the different machines remained largely the same throughout evolution, maintaining, from frog to humans, its basic property to resist stretching with a force greater than that developed during shortening, as described by the force–velocity relation of muscle contractile component.

The finding that the landing–takeoff asymmetry of the animals of this study takes place always in one direction (i.e. with *t*_push_ > *t*_brake_), never the reverse, in spite of the diverse geometries of the lever systems involved, body mass and step frequencies, strongly suggests that the different machines are used to comply with the asymmetric response of their motor during negative and positive work performance. This requirement results in a trend of the *E*_cm_ curve (rounded attainment of plateau and sharp departure from plateau) that is very similar in the 7 kg turkey of figure 1 running at 12 km h^−1^, a 73 kg human running at 13.5 km h^−1^(see fig. 1 of Cavagna 2006), in spite of more than a tenfold difference attained by *E*_cm_ during the step (approx. 40 J in the turkey and 600 J in the human) and of the striking difference of the two locomotors' machines.

### (b) Factors affecting the elastic storage mechanism

In human running the landing–takeoff asymmetry (i.e. the ratio *t*_push_/*t*_brake_) decreases with speed: above 14 km h^−1^ *t*_push_∼*t*_brake_, as expected in an elastic rebound (Cavagna 2006). This finding has been explained as an increase of muscle activation with speed privileging the role of tendons relative to muscle within muscle–tendon units. If muscle activation is so high that muscle does not yield during stretching and is held isometric, as some studies suggest (Roberts *et al*. 1997; Biewener *et al*. 1998), the whole of the length change will be taken up by the tendon, the response of the muscle–tendon unit will approach that of an elastic structure and the landing–takeoff asymmetry would disappear. This mechanism may conveniently apply to kangaroo hopping, which approaches an elastic rebound (i.e. *t*_push_∼*t*_brake_), with most of the length change taken up by tendons (Biewener *et al*. 1998). This is consistent with the high force that must be developed by the muscle to attain the high upward acceleration we measured in these animals (table 1). Furthermore, the efficiency of external work production in kangaroos (Cavagna *et al*. 1977) increases with speed, suggesting an improved elastic storage and recovery as the force exerted on the ground increases.

It has been shown that force enhancement following muscle stretch has a transient character—that is, it disappears rapidly at the end of stretching and, with it, the elastic energy stored within tendons and other undamped elastic elements (Cavagna *et al*. 1986). This transient character of elastic storage and recovery may contribute to the observed decrease of the landing–takeoff asymmetry with running speed in humans.

However, important exceptions indicate that other factors must be taken into account when comparing different groups of subjects. For example, the landing–takeoff asymmetry is greater in the springhare than in the kangaroo in spite of a similarly high upward acceleration (table 1). On the other hand, the landing–takeoff asymmetry is almost nil in the rams, which show the lowest upward acceleration (table 1).

One factor, found in humans, is age: during running the landing–takeoff asymmetry is greater in old subjects than in young subjects, suggesting a less elastic rebound in the elderly (Cavagna *et al*. 2008).

Another factor is size. Data in table 1 suggest that the landing–takeoff asymmetry is greater in small than in large animals. The hypothesis that small animals are unable to use an elastic storage mechanism during the bouncing step as efficiently as large animals do was put forward on the basis that the efficiency of the transformation of metabolic energy into mechanical work is less in small than in large animals (Heglund *et al*. 1982).

The efficiency of the transformation of metabolic energy into external work is given in table 1 for some of the animals (mean of the lowest and highest values of the curves in fig. 10 of Cavagna *et al*. 1977). It can be seen that a larger landing–takeoff asymmetry in the smaller animals (i.e. a greater ratio *t*_push_/*t*_brake_), is associated with a lower metabolic efficiency, supporting the hypothesis that the landing–takeoff asymmetry is a measure of a lack of elasticity in the bouncing step. Both metabolic inefficiency and landing–takeoff asymmetry independently show that elastic energy storage is less efficient in small animals.

It has been suggested that the elastic storage mechanism is lower in small animals because their tendons are relatively thicker than those of large animals. This was measured by comparing small kangaroo rats with large kangaroos (Biewener *et al*. 1981) and calculated from measurements of muscle and tendon dimensions in kangaroos (Bennett & Taylor 1995) and quadrupedal mammals (Alexander *et al*. 1981; Pollock & Shadwick 1994). This hypothesis is consistent with the present findings, showing that the landing–takeoff asymmetry (i.e. the deviation from an elastic bounce) is associated in the smaller animals with a greater stiffness of the system (table 1). The greater mass-specific vertical stiffness in small animals implies a greater natural frequency of the bouncing system *f*_s_ = (1/2π)√*k*/*M*_b_, which in turn is bound to a greater step frequency (table 1). More steps (i.e. a greater step frequency) are required to cover a given distance in the animals of smaller dimensions. This requires a greater frequency of the bouncing system (i.e. a greater mass-specific vertical stiffness), with the drawback of a less efficient elastic rebound, resulting in a greater landing–takeoff asymmetry.

## References

[RSPB20091317C1] Alexander R. McN. 2002 Tendon elasticity and muscle function. Comp. Biochem. Physiol. 133A, 1001–1011 10.1016/s1095-6433(02)00143-5 12485689

[RSPB20091317C2] Alexander R. McN. Jayes A. S. Maloiy G. M. O. Wathuta E. M. 1981 Allometry of the leg muscles of mammals. J. Zool. 194, 539–552

[RSPB20091317C3] Bennett M. B. Taylor G. C. 1995 Scaling of elastic strain energy in kangaroos and the benefits of being big. Nature 378, 56–59 (doi:10.1038/378056a0) 7477284 10.1038/378056a0

[RSPB20091317C4] Biewener A. A. Alexander R. McN. Heglund N. C. 1981 Elastic energy storage in the hopping of kangaroo rats (*Dipodomys spectabilis*). J. Zool. 195, 369–383 (doi:10.1111/j.1469-7998.1981.tb03471.x)

[RSPB20091317C5] Biewener A. A. Konieczynski D. D. Baudinette R. V. 1998 *In vivo* muscle force–length behavior during steady-speed hopping in tammar wallabies. J. Exp. Biol. 201, 1681–1694 9576879 10.1242/jeb.201.11.1681

[RSPB20091317C6] Blickhan R. 1989 The spring-mass model for running and hopping. J. Biomech. 22, 1217–1227 (doi:10.1016/0021-9290(89)90224-8) 2625422 10.1016/0021-9290(89)90224-8

[RSPB20091317C7] Carrier D. R. Heglund N. C. Earls K. D. 1994 Variable gearing during locomotion in the human musculoskeletal system. Science 265, 651–653 (doi:10.1126/science.8036513) 8036513 10.1126/science.8036513

[RSPB20091317C8] Cavagna G. A. 1975 Force platforms as ergometers. J. Appl. Physiol. 39, 174–179 1150585 10.1152/jappl.1975.39.1.174

[RSPB20091317C9] Cavagna G. A. 2006 The landing–take-off asymmetry in human running. J. Exp. Biol. 209, 4051–4060 (doi:10.1242/jeb.02344) 17023599 10.1242/jeb.02344

[RSPB20091317C10] Cavagna G. A. Saibene F. Margaria R. 1964 Mechanical work in running. J. Appl. Physiol. 19, 249–256 14155290 10.1152/jappl.1964.19.2.249

[RSPB20091317C11] Cavagna G. A. Heglund N. C. Taylor C. R. 1977 Mechanical work in terrestrial locomotion: two basic mechanisms for minimizing energy expenditure. Am. J. Physiol. 233, R243–R261 411381 10.1152/ajpregu.1977.233.5.R243

[RSPB20091317C12] Cavagna G. A. Mazzanti M. Heglund N. C. Citterio G. 1986 Mechanical transients initiated by ramp stretch and release to P_0_ in frog muscle fibers. Am. J. Physiol. 251, C571–C579 3490184 10.1152/ajpcell.1986.251.4.C571

[RSPB20091317C13] Cavagna G. A. Franzetti P. Heglund N. C. Willems P. A. 1988 The determinants of the step frequency in running, trotting and hopping in man and other vertebrates. J. Physiol. 399, 81–92 3404473 10.1113/jphysiol.1988.sp017069 PMC1191653

[RSPB20091317C14] Cavagna G. A. Legramandi M. A. Peyre-Tartaruga L. A. 2008 The landing–take-off asymmetry of human running is enhanced in old age. J. Exp. Biol. 211, 1571–1578 (doi:10.1242/jeb.013805) 18456884 10.1242/jeb.013805

[RSPB20091317C15] Heglund N. C. Fedak M. A. Taylor C. R. Cavagna G. A. 1982 Energetics and mechanics of terrestrial locomotion IV—total mechanical energy changes as a function of speed and body size in birds and mammals. J. Exp. Biol. 97, 57–66 7086351 10.1242/jeb.97.1.57

[RSPB20091317C16] McGowan C. P. Baudinette R. V. Biewener A. A. 2008 Differential design for hopping in two species of wallabies. Comp. Biochem. Physiol. 150A, 151–158 10.1016/j.cbpa.2006.06.018 16861021

[RSPB20091317C17] Pollock C. M. Shadwick R. E. 1994 Allometry of muscle, tendon, and elastic energy storage capacity in mammals. Am. J. Physiol. 266, R1022–R1031 8160851 10.1152/ajpregu.1994.266.3.R1022

[RSPB20091317C18] Roberts T. J. Marsh R. L. Weyand P. G. Taylor C. R. 1997 Muscular force in running turkeys: the economy of minimizing work. Science 275, 1113–1115 (doi:10.1126/science.275.5303.1113) 9027309 10.1126/science.275.5303.1113

